# The Impacts of Dietary Fermented Mao-tai Lees on Growth Performance, Plasma Metabolites, and Intestinal Microbiota and Metabolites of Weaned Piglets

**DOI:** 10.3389/fmicb.2021.778555

**Published:** 2021-11-29

**Authors:** Zhihua Li, Qian Zhu, Md. Abul Kalam Azad, Huawei Li, Pan Huang, Xiangfeng Kong

**Affiliations:** ^1^Hunan Provincial Key Laboratory of Animal Nutritional Physiology and Metabolic Process, National Engineering Laboratory for Pollution Control and Waste Utilization in Livestock and Poultry Production, Institute of Subtropical Agriculture, Chinese Academy of Sciences, Changsha, China; ^2^College of Animal Science and Technology, Nanjing Agricultural University, Nanjing, China

**Keywords:** amino acids, fermented Mao-tai lees, growth performance, intestinal microbiota, weaned piglets

## Abstract

This study investigated the effects of dietary supplementation with fermented Mao-tai lees (FML) on growth performance, plasma metabolites, and intestinal microbiota and metabolites of weaned piglets. A total of 128 *Duroc*×*Landrace*×*Yorkshire* piglets (28-days old) were randomly assigned to one of four groups, feeding a basal diet (control group), a basal diet supplemented with 2, 4 or 6% FML, respectively, for 42days. The results showed that dietary 4% FML supplementation had higher (*p*<0.05) average daily gain (ADG) and plasma triglyceride concentration during days 1–14 of the trial than the other FML supplemented groups. In addition, dietary 2 and 4% FML supplementation increased (*p*<0.05) the ADG during days 15–28 of the trial and plasma total protein concentration on day 42 of the trial compared with the 6% FML supplement. The plasma concentrations of arginine, ethanolamine, histidine, isoleucine, lysine, methionine, proline, taurine, threonine, and tyrosine were increased (*p*<0.05) in the 4% FML group compared with the other three groups on day 14 of the trial. Dietary supplementation with 2–6% FML decreased (*p*<0.05) the plasma urea nitrogen concentration on day 14 of the trial and the abundance of *Escherichia coli* in the colon, and dietary 2 and 4% FML supplementation decreased (*p*<0.05) the abundance of sulfate-reducing bacteria compared with the control group. In the intestinal contents, a higher concentration of FML (6%) supplementation decreased (*p*<0.05) the colonic acetate concentration compared with the control and 2% FML groups, while 4% FML supplementation increased (*p*<0.05) the colonic cadaverine concentration compared with the other three groups. In conclusion, dietary 4% FML supplementation might contribute to the increased amino acids metabolism without affecting the growth performance of weaned piglets. Moreover, dietary 2 and 4% FML supplementation were also beneficial to intestinal health *via* decreasing the abundances of specific pathogens and increasing the concentrations of microbial metabolites in the gut, which provides the theoretical basis and data support for the application of FML in pigs.

## Introduction

To reduce production costs, livestock producers have increased the use of agricultural by-products, including distillers dried grains with soluble (DDGS) of sorghum, wheat, corn, and rye, which can replace some conventional feedstuffs. The DDGS from six different dry grind ethanol plants contains various nutrients, including 7.89–15.10% oil, 28.01–30.03% crude protein (CP), 37.51–48.74% neutral detergent fiber (NDF), 13.92–18.71% acid detergent fiber (ADF), and 3.72–4.59% ash (Singh et al., 2002). Compared with corn and soybean, the DDGS has considerably more fiber which consists predominantly of 35% insoluble dietary fiber (Stein and Shurson, 2009) with low fermentation performance, including arabinoxylans, cellulose, and lignin (Gutierrez et al., 2014). Mao-tai liquor is famous due to its unique production process, impressive liquor quality, and complex aromas (Wang et al., 2016). The production of Mao-tai liquor was 50,235.17tons in Kweichow Moutai Co., Ltd. (2020), Guizhou, China (Annual Report, 2020), and the solid by-products (including lees) are estimated to be about 150,700tons. After the processing and production of liquor, a large amount of solid by-products remained unused, which can cause resource waste and environmental pollution. Therefore, the comprehensive utilization of Mao-tai lees (a common DDGS) in animal feed is conducive to the sustainable development of the wine industry and solves the problem of feedstuff shortage in animal husbandry.

Piglets are vulnerable to physiological, nutritional, and environmental weaning stressors due to the transportation, mixing with other littermates, separation from the sow’s milk, and changes to solid feed and ambient temperature (Tang et al., 2009; Azad et al., 2021). These abrupt changes often cause intestinal microbiota imbalance, which has been related to decreased growth rate, changes in gut morphology and microbial populations, and increased susceptibility to scouring and disease (Reilly et al., 2008). Previous studies indicated that increasing DDGS inclusion in diets resulted in the reduction of body gain to feed intake of weaned piglets, which might be due to the increasing unfermented DDGS inclusion reached to a physical limitation of the gut size, or by decreasing the density and altering the taste or smell of the diet, thus reduced the diet palatability (Avelar et al., 2010). Therefore, it is important to improve the digestibility and nutritional value of DDGS. Fermentation is a practical way to increase cellulose and hemicellulose degradation (Yang et al., 2012), lower phytic acid, and enhance protein digestibility, so fermentation of DDGS may facilitate higher inclusion levels in animal diets, especially for mono-gastric animals (Lamsal et al., 2012). Yeasts, such as *Saccharomyces boulardii,* have increasingly been used for fermenting DDGS, providing vital evidence of its efficacy as an adjuvant agent to treat diarrhea and prevent antibiotic-associated complications (Buts, 2009). Thus, microbial fermentation can improve the nutritional values and functional effects of DDGS.

Previous studies mostly focused on the effects of dietary supplementation with DDGS and fermented DDGS on the growth performance and digestibility of animals (Stein and Shurson, 2009; Gutierrez et al., 2014). However, the effects of dietary supplementation with DDGS on plasma metabolites and intestinal microbiota and their metabolites have not been elucidated yet. Our previous studies showed that supplementing 15% fermented Mao-tai lees (FML) to growing-finishing pigs’ diets could improve the gut health *via* increasing the abundance of putative beneficial microbiota and straight-chain fatty acid concentrations, and decreasing the abundances of potential pathogens in the colon (Li et al., 2019). Therefore, we hypothesized that the FML could be used as a cost-effective alternative for feedstuff of weaned piglets owing to its beneficial to plasma and intestinal metabolites and intestinal microbiota. Thus, the present study determined the effects of dietary FML supplementation on growth performance, plasma metabolites, and intestinal microbiota and metabolites of weaned piglets.

## Materials and Methods

### Preparation of FML

The FML was prepared by the Road Biological Environmental Co., Ltd., Sichuan, China. After sterilization, Mao-tai liquor lees containing 55% water were mixed with 5‰ *Saccharomyces cerevisia*e, 3‰ *Aspergillus niger*, and 0.6‰ *Bacillus subtilis*. The mixture was incubated at 32°C for 36h under ventilation and at 32°C for 12h under static and anaerobic conditions. Then, the incubated mixture was added into 0.5‰ acid protease and 0.5‰ neutral protease and further incubated at pH 5.0 and 60°C for 12h. After fermentation, the fermented products were dried using a fluidized bed dryer at 40°C, pulverized, screened through a 0.45-mm mesh, and packed for use. The determined nutrient levels (%) of FML based on dry matter content (92.97%) were as follows: ash, 9.28; CP, 23.96; ether extract (EE), 5.39; crude fiber (CF), 17.67; ADF, 38.06; NDF, 47.28; Ca, 0.53; P, 0.55; and gross energy (GE), 18.29MJ/Kg (Li et al., 2019).

### Animals, Housing, and Treatment

A total of 128 *Duroc*×*Landrace*×*Yorkshire* piglets weaned at 21-day old were used in this study. After a 7-day adaptation period, all piglets at 28-day old with 7.94±0.12kg of average body weight (BW) were randomly assigned to one of four groups. The piglets were fed a basal diet (control group), a basal diet supplemented with 2, 4, or 6% FML, respectively. The basal diet (Supplementary Table 1) was formulated to meet the National Research Council (2012) nutrient requirements for weaned piglets. Each group had eight replicates (pens) with four piglets per replicate. The piglets were housed individually in an environmentally controlled room (23±2°C) with a nursery facility with hard plastic completely slotted flooring. The piglets were fed twice daily (at 8:00 and 16:00), and water was available *ad libitum*. Each pen was equipped with a stainless-steel feeder and a nipple drinker. The FML was accurately weighed and fully mixed with the basal feed.

### Growth Performance

Piglet BW was measured on days 14, 28, and 42 of the trial. The feed intake and number of piglets with diarrhea were recorded daily. Average daily gain (ADG), average daily feed intake (ADFI), and the ratio of feed intake to BW gain (F/G) were calculated.

### Sample Collection and Preparation

Blood samples (5ml) were collected from the precaval vein on days 14, 28, and 42 of the trial, respectively. Plasma was obtained by centrifugation at 3,000×*g* and 4°C for 10min and then stored at −80°C until analysis. At the end of the trial, all the animals were electrically stunned (120V, 200HZ), exsanguinated, and eviscerated for sample collection. The contents of the ileum (10cm above the ileo-cecal junction) and colon (middle portion) were collected and divided into two aliquots. One aliquot of the contents (1g) was immediately snap-frozen in liquid nitrogen and then stored at −80°C for analysis of microbial DNA. The other portion (approximately 20g) was stored at −20°C to assay the concentrations of short-chain fatty acids (SCFAs) and bioamines (only in the colon).

### Analysis of Plasma Metabolites

Plasma concentrations of urea nitrogen (UN), total protein (TP), total cholesterol (TC), triglyceride (TG), high-density lipoprotein-cholesterol (HDL-C), and low-density lipoprotein-cholesterol (LDL-C) were analyzed using a Roche automatic biochemical analyzer (Cobas c311, F. Hoffmann-La Roche Ltd., Basel, Switzerland) and commercial kits (F. Hoffmann-La Roche Ltd., Basel, Switzerland) according to the manufacturers’ instructions, as well as the plasma activities of alkaline phosphatase (ALP), aspartate aminotransferase (AST), and alanine aminotransferase (ALT; Kong et al., 2007).

Plasma samples (1.00ml) were blended with the same volume of 8% 5-sulfosalicylic acid for 30min and then centrifuged at 12,000×*g* for 10min at 4°C. The supernatant was filtered through a 0.45-μm membrane for free amino acids (AAs) analysis using an auto amino acid analyzer (L8800, Hitachi, Tokyo, Japan; Hu et al., 2019).

### Determination of Microbiota Abundances in the Ileal and Colonic Contents

The abundances of the ileal and colonic microbiota were analyzed as previously described (Su et al., 2018). Briefly, the total microbial DNA was extracted and purified by a QIAamp DNA Stool Kit (Qiagen, Hilden, Germany). The specific primers (Supplementary Table 2) of targeted microbiota were used to amplify the targeted gene fragments. The recombinant plasmid vector of targeted microbiota was constructed and cloned according to the pMDTM19-T vector cloning kit (TaKaRa Biotechnology, Dalian, China) instructions. Quantitative real-time polymerase chain reaction (qPCR) was performed using an SYBR Green detection kit (Thermo Fisher Scientific, Waltham, MA) to determine the abundances of general microbial DNA from the intestinal contents and above recombinant DNA using a Lightcycler 480II instrument (Applied Biosystems; Su et al., 2018). The standard curves for all determined microbiota were constructed based on the recombinant DNA of representative species. The results are presented as the gene copies of microbial DNA of the intestinal contents.

### Determination of Microbial Metabolites

The ileal and colonic SCFAs, including acetate, propionate, butyrate, isobutyrate, valerate, and isovalerate, were analyzed according to the method described by Zhou et al. (2014). The contents of cadaverine, phenylethylamine, putrescine, tryptamine, tyramine, spermidine, and 1,7-heptyl diamine in the colonic contents were measured as described previously (Ji et al., 2018), using an Agilent 6,890 gas chromatography (Agilent Technologies, Inc., Palo Alto, CA, United States).

### Statistical Analysis

Statistical analysis was performed using SAS software version 9.2. The normal distribution and homogeneity of variance of data were evaluated by the Shapiro–Wilk method and Levene method. Then, all data were subjected to one-way ANOVA with Duncan’s multiple range test. The statistical data are expressed with means and pooled standard error of the means (SEM) among the four groups. Statistical significance was defined as *p*<0.05. Probability values between 0.05 and 0.10 were considered to be trends.

## Results

### Effects of Dietary FML Supplementation on Growth Performance and Diarrhea Rate of Weaned Piglets

To evaluate the effects of FML on the growth performance of weaned piglets, the BW, ADFI, and F/G were measured at different stages of the trial. As shown in Table 1, there were no significant differences (*p*>0.05) in the initial BW, final BW, ADFI, and F/G among the four groups. The 4% FML group had higher (*p*<0.05) ADG during days 1–14 of the trial than the other FML supplemented groups. Moreover, the pigs supplemented with 2 and 4% FML during days 15–28 of the trial had higher (*p*<0.05) ADG than the 6% FML group. There were no significant differences (*p*>0.05) in the diarrhea rate of piglets among the four groups during days 1–14, 15–28, 29–42, and 1–42 of the trial (Supplementary Table 3).

**Table 1:** Effects of dietary supplementation with fermented Mao-tai lees (FML) on growth performance of weaned piglets.

Items	Control	Dietary FML level			SEM	*p* values
		2%	4%	6%		
Initial BW (kg)	7.93	7.93	7.95	7.95	0.12	1.00
Final BW (kg)	26.46	26.93	27.02	25.95	0.44	0.82
ADFI (gd^−1^)						
Days 1–14 of the trial	448.22	448.45	448.31	446.69	2.28	0.99
Days 15–28 of the trial	909.13	946.80	891.32	913.65	12.76	0.50
Days 29–42 of the trial	1204.95	1135.23	1224.21	1181.32	21.91	0.54
Days 1–42 of the trial	847.01	866.74	852.26	847.22	5.99	0.63
ADG (gd^−1^)						
Days 1–14 of the trial	256.67^ab^	241.63^b^	267.08^a^	238.73^b^	3.64	<0.01
Days 15–28 of the trial	510.34^ab^	519.87^a^	519.39^a^	488.91^b^	4.50	0.04
Days 29–42 of the trial	595.76	557.44	583.63	558.93	9.18	0.38
Days 1–42 of the trial	439.57	452.42	452.02	434.57	3.51	0.19
F/G (gg^−1^)						
Days 1–14 of the trial	1.82	1.86	1.79	1.84	0.02	0.38
Days 15–28 of the trial	1.79	1.82	1.78	1.87	0.03	0.64
Days 29–42 of the trial	2.04	2.03	2.08	2.12	0.02	0.82
Days 1–42 of the trial	1.94	1.89	1.92	2.00	0.02	0.15

Data are presented as means with pooled standard error of the means (SEM; *n*=8). ^a, b^Mean values within a row with different superscript letters were significantly different (*p*<0.05). ADFI, average daily feed intake; ADG, average daily gain; BW, body weight; and F/G, ratio of feed to gain.

### Effects of Dietary FML Supplementation on Plasma Biochemical Parameters of Weaned Piglets

The plasma biochemical indicators of weaned piglets are shown in Table 2. On day 14 of the trial, the plasma UN concentration of the FML groups (2, 4, and 6%) was lower (*p*<0.05) than the control group. The plasma TG concentration of the 4% FML group was higher (*p*<0.05) than the 2 and 6% FML groups. On day 28 of the trial, the plasma LDL-C concentration of the 4% FML group was higher (*p*<0.05) than the 6% FML group. The plasma TG concentration of the 4 and 6% FML groups was higher (*p*<0.05) than the control group. Moreover, dietary FML supplementation had a trend to decrease (*p*=0.05) the plasma TP concentration. On day 42 of the trial, the plasma LDL-C concentration of the 6% FML group was higher (*p*<0.05) than the 2% FML and control groups. The plasma TP concentration of the 6% FML group was lower (*p*<0.05) than the other three groups. In addition, the plasma TC concentration of the 4 and 6% FML groups had an increasing trend (*p*=0.06) compared with the control and 2% FML groups. However, there were no significant differences (*p*>0.05) in plasma levels in the plasma concentrations of ALP, ALT, AST, and HDL-C during the entire trial period among the four groups.

**Table 2:** Effects of dietary supplementation with fermented Mao-tai less (FML) on plasma biochemical parameters in weaned piglets.

Items	Day of the trial	Control	Dietary FML level			SEM	*p* values
			2%	4%	6%		
ALP (UL^−1^)	14	509.50	463.00	425.75	357.38	26.00	0.21
	28	435.88	353.50	405.00	385.63	21.20	0.59
	42	241.33	245.17	279.83	274.00	12.78	0.65
ALT (UL^−1^)	14	47.88	56.75	45.75	52.75	2.13	0.26
	28	57.25	47.25	55.13	53.75	2.17	0.41
	42	39.33	41.17	42.17	42.50	2.44	0.33
AST (UL^−1^)	14	71.63	76.75	87.50	86.25	5.43	0.71
	28	76.50	67.38	68.38	61.38	3.10	0.40
	42	61.33	51.50	61.67	69.50	3.30	0.30
UN (mmolL^−1^)	14	3.22^a^	2.15^b^	2.23^b^	2.31^b^	0.13	<0.01
	28	2.52	2.66	2.16	2.11	0.12	0.27
	42	2.62	3.19	2.95	2.24	0.17	0.25
HDL-C (mmolL^−1^)	14	0.61	0.68	0.67	0.62	0.02	0.72
	28	0.69	0.76	0.79	0.70	0.02	0.36
	42	0.70	0.72	0.93	0.80	0.04	0.12
LDL-C (mmolL^−1^)	14	0.91	0.82	0.80	0.97	0.04	0.34
	28	1.02^ab^	1.04^ab^	1.17^a^	0.91^b^	0.03	0.04
	42	1.00^b^	0.88^b^	1.07^ab^	1.36^a^	0.06	0.02
TC (mmolL^−1^)	14	1.66	1.64	1.64	1.70	0.05	0.97
	28	1.84	1.90	2.07	1.77	0.05	0.11
	42	1.80	1.69	2.07	2.21	0.08	0.06
TG (mmolL^−1^)	14	0.39^ab^	0.32^b^	0.48^a^	0.36^b^	0.02	0.04
	28	0.40^b^	0.44^ab^	0.63^a^	0.58^a^	0.03	0.04
	42	0.27	0.40	0.39	0.26	0.03	0.29
TP (gL^−1^)	14	45.91	39.88	39.68	39.93	1.28	0.24
	28	52.64	42.96	42.53	45.83	1.35	0.05
	42	55.52^a^	57.72^a^	53.10^a^	44.98^b^	1.46	<0.01

Data are presented as means with pooled SEM (*n*=8). ^a, b^Mean values within a row with different superscript letters were significantly different (*p*<0.05). ALP, alkaline phosphatase; ALT, alanine aminotransferase; AST, aspartate transaminase; UN, urea nitrogen; HDL-C, high-density lipoprotein-cholesterol; LDL-C, low-density lipoprotein-cholesterol; TC, total cholesterol; TG, triglyceride; and TP, total protein.

### Effects of Dietary FML Supplementation on Plasma-Free AAs in Weaned Piglets

We further investigated the effects of FML supplementation on piglet’s plasma-free AAs profiles. As shown in Table 3, the plasma concentrations of arginine (Arg), ethanolamine (EOHNH2), histidine (His), isoleucine (Ile), lysine (Lys), methionine (Met), proline (Pro), taurine (Tau), threonine (Thr), and tyrosine (Tyr) in the 4% FML group were higher (*p*<0.05) compared with the other three groups on day 14 of the trial. In addition, the plasma concentration of sarcosine (Sar) was higher (*p*<0.05) in the 4 and 6% FML groups than the control and 2% FML groups. On day 28 of the trial, the plasma concentrations of cysteine (Cys; *p*<0.05) and Tau (*p*=0.08) of the 4% FML group were higher than the other three groups. Moreover, on day 42 of the trial, the plasma concentration of citrulline (Cit) of the 2 and 6% FML groups was lower (*p*<0.05), as well as the plasma concentration of EOHNH2 of the three FML groups, while the plasma Pro concentration of the 2% FML group was higher (*p*<0.05), when compared with the control group.

**Table 3:** Effects of dietary supplementation with fermented Mao-tai less (FML) on plasma concentration of free amino acids in weaned piglets.

Items (μmolL^−1^)	Day of the trial	Control	Dietary FML level			SEM	*p* values
			2%	4%	6%		
Ala	14	611.14	745.97	816.85	779.94	31.57	0.10
	28	695.50	676.26	777.23	759.27	21.86	0.30
	42	381.94	590.29	574.96	603.71	34.22	0.08
Arg	14	101.06^b^	80.45^b^	150.10^a^	108.69^b^	6.83	<0.01
	28	124.91	123.27	143.54	113.44	8.05	0.63
	42	103.63	91.01	83.61	91.90	4.07	0.38
Asp	14	32.80	31.37	41.39	30.91	1.77	0.14
	28	34.95	32.08	41.26	35.58	2.52	0.65
	42	19.48	29.35	25.16	27.16	1.58	0.13
Cit	14	84.51	76.58	77.39	77.67	2.88	0.77
	28	70.08	72.65	74.10	76.54	2.07	0.75
	42	70.94^a^	57.69^bc^	68.46^ab^	54.33^c^	2.41	0.03
Cys	14	16.42	10.76	12.81	14.46	1.03	0.24
	28	13.81^b^	11.76^b^	23.55^a^	13.15^b^	1.7	0.05
	42	14.13	6.53	5.77	11.96	1.53	0.13
EOHNH2	14	2.94^b^	3.12^b^	5.60^a^	1.86^b^	0.43	0.02
	28	2.69	2.06	4.79	3.83	0.46	0.15
	42	4.79^a^	0.92^b^	1.54^b^	0.78^b^	0.42	<0.01
Glu	14	417.43	402.24	519.13	472.22	24.82	0.33
	28	361.24	372.61	444.16	373.75	18.4	0.38
	42	232.74	318.50	274.69	335.74	20.57	0.30
Gly	14	1265.73	1588.42	1337.18	1299.56	63.89	0.27
	28	1271.19	1384.78	1280.59	1222.47	44.88	0.65
	42	870.66	1026.73	993.20	972.76	48.75	0.71
His	14	11.90^b^	11.17^b^	20.18^a^	12.15^b^	1.18	0.01
	28	28.19	24.86	23.42	25.65	1.06	0.47
	42	21.19	20.60	19.18	20.35	0.66	0.76
Ile	14	113.19^b^	107.60^b^	144.31^a^	116.61^b^	4.74	0.02
	28	106.47	110.74	112.05	97.80	2.81	0.30
	42	88.34	99.06	91.50	80.23	3.57	0.35
Leu	14	115.68	107.03	139.81	115.99	5.15	0.13
	28	189.22	182.89	197.34	170.43	5.21	0.34
	42	175.80	198.00	190.53	158.17	6.59	0.17
Lys	14	187.54^b^	199.89^b^	303.36^a^	207.59^b^	13.82	<0.01
	28	300.40	275.16	308.42	310.36	13.14	0.78
	42	155.73	187.96	136.45	168.16	12.42	0.53
Met	14	51.92^b^	44.14^b^	87.93^a^	59.79^b^	4.26	<0.01
	28	58.68	55.81	53.15	53.12	2.22	0.80
	42	31.59	30.43	34.94	28.46	1.46	0.50
Orn	14	116.66	106.14	140.83	115.56	5.76	0.18
	28	137.32	147.60	130.51	122.03	4.68	0.30
	42	81.60	79.50	77.59	75.28	3.54	0.95
Phe	14	57.36^ab^	44.83^b^	68.82^a^	65.25^a^	2.85	0.01
	28	78.74	65.12	71.97	59.25	2.73	0.06
	42	64.35	62.88	62.85	60.44	2.51	0.97
Pro	14	254.18^b^	265.42^b^	328.20^a^	270.46^b^	9.05	0.01
	28	294.70	314.03	305.90	334.79	7.93	0.34
	42	202.03^b^	281.91^a^	258.88^ab^	232.56^ab^	11.12	0.04
Sar	14	30.31^b^	34.36^b^	47.67^a^	45.99^a^	2.24	0.01
	28	41.99	44.90	47.13	48.82	1.50	0.42
	42	38.40	42.35	46.41	39.26	2.30	0.62
Ser	14	198.05	247.47	267.35	244.74	11.65	0.19
	28	184.80	199.18	204.12	201.97	7.45	0.81
	42	120.94	138.06	125.36	129.5	4.42	0.58
Tau	14	95.20^b^	92.31^b^	164.05^a^	104.53^b^	7.83	<0.01
	28	122.77	100.68	142.94	91.77	7.77	0.08
	42	78.22	56.44	62.13	62.05	5.57	0.56
Thr	14	79.10^bc^	68.20^c^	215.14^a^	123.57^b^	13.48	<0.01
	28	85.69	86.64	86.31	79.46	4.50	0.94
	42	50.83	52.43	68.66	52.73	3.29	0.17
Tyr	14	61.98^b^	54.70^b^	97.12^a^	67.53^b^	4.51	<0.01
	28	94.45	84.90	87.79	93.09	3.80	0.81
	42	68.39	74.65	59.35	54.32	3.23	0.12
Val	14	127.01	98.00	130.39	110.07	6.05	0.20
	28	159.69	150.71	157.95	155.17	5.09	0.94
	42	168.13	170.66	156.72	152.32	6.71	0.76

Data are presented as means with pooled SEM (*n*=8). ^a–c^Mean values within a row with different superscript letters were significantly different (*p*<0.05). Ala, alanine; Arg, arginine; Asp, aspartic acid; Cit, citrulline; Cys, cysteine; EOHNH2, ethanolamine; Glu, glutamic acid; Gly, glycine; His, histidine; Ile, isoleucine; Leu, leucine; Lys, lysine; Met, methionine; Orn, ornithine; Phe, phenylalanine; Pro, proline; Sar, sarcosine; Ser, serine; Tau, taurine; Thr, threonine; Tyr, tyrosine; and Val, valine.

### Effects of Dietary FML Supplementation on Microbiota Abundances in the Ileal and Colonic Contents of Weaned Piglets

The microbiota abundances in the ileal and colonic contents of weaned piglets are shown in Table 4. There were no significant differences (*p*>0.05) in the abundances of *Clostridium cluster* IV, *Bacteroidetes*, *Lactobacillus*, *Escherichia coli (E. coli),* and total bacteria in the ileal contents were observed among the four groups. The relative abundance of *Firmicutes* in the 6% FML group was lower (*p*<0.05) compared with the control group. Meanwhile, the ratio of *Firmicutes* to *Bacteroidetes* (F/B) in the 4 and 6% FML groups was lower (*p*=0.07) compared with the control and 2% FML groups. Dietary 4% FML supplementation increased (*p*<0.05) the abundance of sulfate-reducing bacteria compared with the control and 2% FML groups.

**Table 4:** Effects of dietary supplementation with Fermented Mao-tai Lees (FML) on microbiota abundance in ileal and colonic contents of weaned piglets.

Items (copies g^−1^)	Control	Dietary FML level			SEM	*p* values
		2%	4%	6%		
**Ileum**
*Clostridium cluster* IV (10^5^)	2.01	2.00	1.97	0.92	0.31	0.51
*Bacteroidetes* (10^6^)	3.80	1.85	2.79	3.07	0.60	0.76
*Escherichia coli* (10^6^)	5.43	1.09	4.81	0.43	1.00	0.18
*Firmicutes* (10^8^)	12.25^a^	3.78^ab^	4.22^ab^	1.17^b^	1.60	0.04
F/B	251.15	243.86	74.44	40.37	38.16	0.07
*Lactobacillus* (10^6^)	15.06	16.09	9.50	0.34	3.18	0.29
Sulfate-reducing bacteria (10^4^)	1.13^b^	0.88^b^	4.60^a^	2.37^ab^	0.51	0.04
Total bacteria (10^9^)	28.35	5.39	40.13	1.93	7.28	0.18
**Colon**						
*Clostridium cluster* IV (10^7^)	23.42	34.78	7.71	27.90	4.42	0.14
*Bacteroidetes* (10^10^)	2.51	3.56	2.58	3.81	0.33	0.46
*Escherichia coli* (10^6^)	33.06^a^	6.94^b^	3.25^b^	1.71^b^	3.99	0.03
*Firmicutes* (10^9^)	9.91	14.42	18.85	18.19	2.03	0.48
F/B	0.45^b^	0.38^b^	0.96^a^	0.59^b^	0.07	<0.01
*Lactobacillus* (10^6^)	18.18	3.19	1.44	6.91	2.45	0.15
Sulfate-reducing bacteria (10^4^)	9.81^a^	1.27^b^	2.01^b^	5.81^ab^	1.14	0.02
Total bacteria (10^11^)	8.59	10.70	4.43	9.88	1.71	0.58

Data are presented as means with pooled SEM (*n*=8). ^a, b^Mean values within a row with different superscript letters were significantly different (*p*<0.05). F/B, Firmicutes/Bacteroidetes.

In the colonic contents, dietary 4% FML supplementation increased (*p*<0.05) the F/B ratio compared with the other three groups. In addition, the relative abundance of *E. coli* in the FML groups (2, 4, and 6%) and the relative abundance of sulfate-reducing bacteria in the 2 and 4% FML groups were lower (*p*<0.05) compared with the control group.

### Effects of Dietary FML Supplementation on Intestinal Concentrations of SCFAs and Bioamines of Weaned Piglets

Ileal and colonic SCFAs concentrations are presented in Table 5. The piglets fed with 6% FML had lower (*p*<0.05) colonic acetate concentration than the control and 2% FML groups. A higher (*p*=0.09) straight-chain fatty acid concentration in the colon was observed in the 2% FML group compared with the control and 6% FML groups. However, there were no significant differences (*p*>0.05) in the concentrations of other SCFAs among the four groups.

**Table 5:** Effect of dietary supplementation with fermented Mao-tai less (FML) on intestinal SCFA contents of weaned piglets.

Items	Control	Dietary FML level			SEM	*p* values
		2%	4%	6%		
**Ileum (mgg** ^**−1**^ **)**						
Acetate	0.29	0.16	0.40	0.30	0.05	0.33
Propionate	0.02	0.01	0.02	0.02	0.00	0.64
Butyrate	0.03	0.01	0.04	0.03	0.01	0.53
Isobutyrate	0.006	0.009	0.007	0.006	0.00	0.92
Valerate	0.009	0.003	0.007	0.007	0.00	0.43
Isovalerate	0.008	0.01	0.009	0.014	0.00	0.19
Total straight-chain fatty acids	0.35	0.18	0.46	0.36	0.05	0.33
Total BCFA	0.01	0.02	0.02	0.03	0.00	0.57
Total SCFA	0.37	0.20	0.33	0.38	0.04	0.36
**Colon (mgg** ^**−1**^ **)**						
Acetate	3.48^a^	3.89^a^	3.44^ab^	2.94^b^	0.11	0.01
Propionate	1.76	2.33	1.86	1.83	0.11	0.18
Butyrate	1.01	1.29	1.28	1.15	0.07	0.40
Isobutyrate	0.11	0.11	0.14	0.11	0.01	0.16
Valerate	0.19	0.26	0.25	0.27	0.02	0.64
Isovalerate	0.18	0.16	0.23	0.18	0.01	0.28
Total straight-chain fatty acids	6.35	7.46	6.72	5.95	0.23	0.09
Total BCFA	0.28	0.27	0.37	0.30	0.02	0.24
Total SCFA	6.69	7.35	7.09	5.90	0.24	0.14

Data are presented as means with pooled SEM (*n*=8). ^a, b^Mean values within a row with different superscript letters were significantly different (*p*<0.05). BCFA, branched-chain fatty acid; SCFA, short-chain fatty acid.

Colonic bioamine concentrations of piglets are presented in Table 6. The 4% FML group had a higher (*p*<0.05) cadaverine concentration in the colonic contents than the other three groups. There were no significant differences (*p*>0.05) in the concentrations of other bioamines among the four groups.

**Table 6:** Effects of dietary supplementation with fermented Mao-tai lees (FML) on colonic bioamine concentrations of weaned piglets.

Items (μgg^−1^)	Control	Dietary level of FML			SEM	*P* values
		2%	4%	6%		
Cadaverine	8.51^b^	11.91^b^	30.52^a^	10.09^b^	2.53	0.01
1,7-heptanediamine	1.27	0.63	0.94	0.96	0.16	0.53
Phenylethylamine	2.66	1.64	1.15	1.43	0.24	0.12
Putrescine	17.65	15.51	15.15	11.72	1.63	0.64
Spermidine	19.54	15.29	21.07	9.53	2.47	0.46
Spermine	1.05	1.06	2.05	0.75	0.27	0.51
Tryptamine	3.34	3.76	2.56	2.29	0.34	0.42
Tyramine	9.41	4.62	3.35	4.16	1.47	0.46
Total bioamine	63.08	51.80	60.76	38.86	5.17	0.36

Data are presented as means with pooled SEM (*n*=8). ^a, b^Mean values within a row with different superscript letters were significantly different (*p*<0.05).

## Discussion

The nutritional values of DDGS may be improved by microbial fermentation or enzymatic degradation. Wiseman et al. (2017) reported that fermented DDGS is beneficial to improve the growth performance in weaned piglets, possibly due to nutrient breakdown and normal development of the gut microbiota. In the present study, dietary FML supplementation regulated plasma-free AAs metabolism and altered intestinal microbiota and metabolites of weaned piglets.

The growth performance of weaned piglets can reflect the subsequent performance and the production profitability to some extent. In the present study, although there were no statistical significances in the growth performance of piglets compared with the control group; however, the dietary supplementation with 4% FML had a higher ADG than the other three groups during days 1–14 of the trial. Several previous studies also showed that dietary DDGS supplementation with enzymes was not effective in improving the growth performance of nursery pigs (Jones et al., 2010; Kerr et al., 2013). However, other studies reported that multi-enzyme blends were tended to improve the growth performance in growing-finishing pigs fed diets with DDGS (Emiola et al., 2009; Li et al., 2012). Therefore, the inconsistency might have resulted from differences in the DDGS source, dietary level of the DDGS used, supplementing dose and types of the enzymes or microorganisms used, age of experimental animals, and the nutrient level of the experimental diet (Swiatkiewicz et al., 2016). The diarrhea rate during the nursery period is an essential indicator of the gut health status of piglets. Our results showed that the FML had no adverse side effects on the piglets’ diarrhea rate. Thus, these findings indicate that the dietary FML could be used in mono-gastric animals because microbial fermentation could improve the quality of protein and decrease the CF in the Mao-tai lees.

Plasma metabolites primarily originate from the digestion and absorption of the gut. The UN and TP play a critical role in transamination and reflect the status of protein synthesis and catabolism (Liu et al., 2015). The UN is the main nitrogenous product of protein, and its concentration reflects the efficiency of protein utilization (Chen et al., 2018). Lower UN concentration indicates a good balance of AAs (Hahn et al., 1995). In the present study, dietary FML supplementation decreased the plasma UN concentration on day 14 of the trial, suggesting that dietary FML improved the utilization of protein (Zhai et al., 2018). In addition, dietary 6% FML supplementation decreased the plasma TP concentration on day 42 of the trial, which may be due to that the relatively high fiber content decreased the digestion and absorption of dietary protein. The ALT and AST are transaminases which play crucial roles in protein and amino acid metabolism, and their plasma activities may increase when tissues damage and dysfunction, particularly in the liver (Canli and Canli, 2015). In the present study, dietary FML supplementation did not affect the plasma ALT and AST activities, suggesting that dietary FML had no detrimental effect on the liver tissue.

The TG plays an important role in nutrient metabolism as an energy source and transporter of dietary fat. There is no apparent relationship between the decrease in LDL-C and the increase in HDL-C (Barter et al., 2010). The LDL can antagonize the quorum sensing system that upregulates the gene expressions required for invasive *Staphylococcus aureus* infection (Peterson et al., 2008). In the present study, dietary 4% FML supplementation increased the plasma TG level on day 14 of the trial and LDL-C level on day 28 of the trial without decreasing the HDL-C level, which may be useful for promoting the ADG and inhibiting pathogenic infection.

Plasma proteins mostly exert their physiological functions in the form of AAs in the body. In the present study, dietary 4% FML supplementation increased the plasma concentrations of Arg, EOHNH2, His, Ile, Lys, Met, phenylalanine, Pro, Tau, Thr, and Tyr on day 14 of the trial. These findings indicated that the AA catabolism was improved by the FML supplementation, which may be due to high levels of acidic protein, cellulose, AAs, and organic acids in the FML. The AAs play important roles in regulating food intake and nutrient metabolism in animals (Wu et al., 2014). Several AAs (e.g., Arg, glutamic acid, glycine, Pro, and leucine) participate in cell signal transmission and metabolic regulation (Wu et al., 2014). The Tau, a sulfated compound synthesized by the eukaryotic host, could increase the ADG and hepatic antioxidant status, while relieve lipopolysaccharide-induced inflammation of broiler chickens (Han et al., 2020). A constant supply of sufficient AAs to living cells from the blood is required to ensure protein accretion in the skeletal muscle of growing pigs (Regmi et al., 2016). This may be one of the reasons why the 4% FML supplementation increased the ADG during days 1–14 of the trial.

In the gut microbiota of pigs, *Firmicutes* and *Bacteroidetes* are the most abundant bacteria (Guo et al., 2008). The piglets with a higher F/B ratio are easier to obtain energy from food (Turnbaugh et al., 2006). The present study showed that dietary 4% FML supplementation significantly increased the F/B ratio in the colon, which may increase the energy utilization of feed. *Clostridium clusters* IV produces butyrate as a result of carbohydrate fermentation (Duytschaever et al., 2012). *Lactobacillus* is a well-known probiotic offering diverse applications. Furthermore, *Lactobacillus plantarum* plays a role in preventing diarrhea, lowering cholesterol, reducing irritable bowel syndrome symptoms, and producing plantaricins (Seddik et al., 2017). In the present study, dietary 2–6% FML supplementation had no significant effect on the abundances of *Clostridium clusters* IV and *Lactobacillus* of the weaned pigs. However, our previous studies showed that dietary 15% FML supplementation could increase the abundance of *Lactobacillus* of the growing-finishing pigs (Li et al., 2019). This discrepancy might be related to the different doses of FML supplementation and the age of pigs.

*E. coli* includes harmless commensal and different pathogenic variants which can cause enteric diseases, such as diarrhea or dysentery, and even lead to extra-intestinal infections (Leimbach et al., 2013). Therefore, lower levels of pathogenic *E. coli* in the gut could reduce the incidence of diarrhea in piglets. Sulfate-reducing bacteria in the large intestine can produce volatile sulfur compounds, such as hydrogen sulfide and methanethiol, which are responsible for fecal odor (Ushid et al., 2002). Sulfate-reducing bacteria are also a potential player in the etiology of intestinal disorders, inflammatory bowel diseases, and colorectal cancer in particular (Carbonero et al., 2012). In the present study, dietary 2 and 4% FML supplementation could decrease amounts of *E. coli* and sulfate-reducing bacteria in the colon. Our previous studies also confirmed that dietary 15% FML supplementation could decrease the abundance of potential pathogen *Escherichia* spp. in pigs at different ages (Li et al., 2019). These findings suggested that dietary FML supplementation, particularly 4% FML, could regulate the balance of intestinal microflora.

The SCFAs, especially butyrate, are mainly produced by microbial fermentation of carbohydrates and non-starch polysaccharides in the large intestine, where the relative abundances of microbiota are greater than those in the small intestine (Le et al., 2016). Similarly, the concentrations of individual and total SCFAs were much lower in the ileum than those in the colon. The SCFAs absorbed in the colon not only serve as an energy source but also play roles in the regulation of the immune system, colonic gene expression, gut motility, and metabolic regulation (Puertollano et al., 2014). Acetate can protect gastrointestinal mucosa from ethanol-induced injuries *via* suppressing gastrointestinal oxidation, inflammation, and apoptosis (Liu et al., 2017). In the present study, dietary 2% FML supplementation improved the levels of acetate and straight-chain fatty acids in the colonic contents than the higher percentage of FML supplementation (6%), which possibly a lower percentage of FML benefits to intestinal health of weaned piglets.

Cadaverine is synthesized from the Lys through decarboxylation by lysine decarboxylase in microbiota and ornithine decarboxylase 1 in mammalian cells (Bekebrede et al., 2020), which is essential for the maintenance of cell growth and macromolecular biosynthesis by interacting with nucleic acids, proteins, and membranes (Millerfleming et al., 2015), and can protect cells from oxidative stress by scavenging superoxide radicals (Kang et al., 2007). In addition, cadaverine can regulate the gut physiology of animals in different ways (Wang et al., 2015). In the present study, dietary 4% FML supplementation increased the colonic cadaverine content of piglets. Bioamine level could be upregulated through the phosphorylation of related enzymes by activating mTORC1 (Bekebrede et al., 2020). Therefore, the present study results suggest that the increased colonic cadaverine level in the dietary 4% FML group may be *via* regulating the mTORC1 pathway. However, the underlying mechanism needs to be further clarified.

## Conclusion

In summary, dietary FML supplementation (2 and 4%) could partly improve plasma-free AA levels of the weaned piglets without affecting the piglets’ growth performance. Furthermore, dietary 4% FML supplementation was more beneficial to intestinal health *via* decreasing the abundances of *E. coli* and sulfate-reducing bacteria, and increasing the concentration of cadaverine in the colon. This study also showed that dietary 4% FML supplementation had more distinct effects than the dietary 2 and 6% FML supplements. Thus, the FML could be used as an effective feed additive, which is not only beneficial to animal health but also resource-conserving and environment-friendly.

## Data Availability Statement

The original contributions presented in the study are included in the article/Supplementary Material, and further inquiries can be directed to the corresponding author.

## Ethics Statement

The animal study was reviewed and approved by the Animal Care and Use Committee of the Institute of Subtropical Agriculture, Chinese Academy of Sciences.

## Author Contributions

ZL and XK conceived and designed the experiment. ZL, QZ, PH, and HL performed the experiment. ZL, QZ, and MA processed the data. ZL prepared and drafted the manuscript. MA and XK revised the manuscript. All authors have read and approved the final manuscript.

## Funding

The present work was jointly supported by the National Natural Science Foundation of China (31772613) and Special Funds for Construction of Innovative Provinces in Hunan Province (2019RS3022).

## Conflict of Interest

The authors declare that the research was conducted in the absence of any commercial or financial relationships that could be construed as a potential conflict of interest.

## Publisher’s Note

All claims expressed in this article are solely those of the authors and do not necessarily represent those of their affiliated organizations, or those of the publisher, the editors and the reviewers. Any product that may be evaluated in this article, or claim that may be made by its manufacturer, is not guaranteed or endorsed by the publisher.
