# Supplementary material for: The Impacts of Dietary Fermented Mao-tai Lees on Growth Performance, Plasma Metabolites, and Intestinal Microbiota and Metabolites of Weaned Piglets
Source: Front Microbiol. 2021 Nov 29;12:778555. doi: 10.3389/fmicb.2021.778555 (PMC8667599; doi:10.3389/fmicb.2021.778555)
Supplement: Supplementary file 1 [file Table_1.DOCX]

**The impacts of dietary fermented Mao-tai lees on growth performance, plasma metabolites, and intestinal microbiota and metabolites of weaned piglets**

**Supplementary Table 1** Composition and nutrient levels of the basal diet (air-dry basis; %).

| Items | Prophase nursery diet ^c^  (7 to 15 kg) | Anaphase nursery diet  (16 to 30 kg) |
| --- | --- | --- |
| Ingredients (%) |  |  |
| Corn | 22.00 | 69.50 |
| Broken rice | 25.00 | — |
| Wheat | 12.00 | — |
| Glucose | 3.00 | — |
| Soybean meal（46% CP） | 10.50 | — |
| Soybean meal（43% CP） | — | 16.00 |
| Extruded soybean | 10.00 | — |
| Fermented soybean meal | 2.50 | 4.00 |
| Soybean protein concentrate | — | 2.00 |
| Imported fish meal | 3.00 | 1.00 |
| Low protein whey powder | 5.00 | — |
| Egg powder | 0.50 | — |
| Wheat bran | — | 2.00 |
| Soybean oil | 1.00 | 1.50 |
| Citric acid | 1.50 | — |
| Premix ^a^ | 4.00 | 4.00 |
| Total | 100.00 | 100.00 |
| Nutrient levels, % | | |
| DE（Kcal/kg） | 3400.00 | 3300.00 |
| CP | 18.02 | 16.59 |
| EE | 4.37 | 4.47 |
| CA | 3.82 | 5.15 |
| CF | 2.31 | 2.76 |
| Ca | 0.80 | 0.77 |
| P | 0.55 | 0.57 |
| AP ^b^ | 0.40 | 0.33 |
| Lys | 1.38 | 1.18 |
| Met | 0.42 | 0.39 |
| Cys | 0.28 | 0.31 |
| Thr | 0.87 | 0.72 |
| Trp | 0.24 | 0.23 |

^a^ The premix provides the following per kilogram of the diet: VA 6 200 IU, VD3 700 IU, VE 88 IU, VK 4.4 mg, VB2 8.8 mg, pantothenic acid 24.2 mg, nicotinic acid 33 mg, choline chloride 330 mg, Fe 145 mg, Cu 10 mg, Zn 100 mg, Mn 40 mg, I 0.3 mg, Se 0.1 mg. ^b^ AP, available P; Data are calculated values. ^c^ Containing 3 kg/T ZnO.

**Supplementary Table 2** Group-specific primer sequences for bacteria.

| Items | Sequence (5'−3') | Product size (bp) |
| --- | --- | --- |
| *Clostridium cluster* IV | F：GCACAAGCAGTGGAGT | 240 |
|  | R：CTTCCTCCGTTTTGTCAA |  |
| *Firmicutes* | F：GGAGYATGTGGTTTAATTCGAAGCA | 126 |
|  | R：AGCTGACGACAACCATGCAC |  |
| *Bacteroidetes* | F：GGARCATGTGGTTTAATTCGATGAT | 126 |
|  | R：AGCTGACGACAACCATGCAG |  |
| *Lactobacillus* | F：AGCAGTAGGGAATCTTCCA | 345 |
|  | R：ATTCCACCGCTACACATG |  |
| *Escherichia coli* | F：CATGCCGCGTGTATGAAGAA | 95 |
|  | R：CGGGTAACGTCAATGAGCAAA |  |
| Sulfate-reducing bacteria | F：TGGCAGATMATGATYMACGG | 387 |
|  | R：GGGCCGTAACCGTCCTTGA |  |
| Total bacteria | F：GTGSTGCAYGGYYGTCGTCA | 123 |
|  | R：ACGTCRTCCMCNCCTTCCTC |  |

**Supplementary Table 3** Effects of dietary supplementation with fermented Mao-tai lees (FML) on diarrhea rates of nursery pigs (%).

| Items | Control | Dietary level of FML | | | SEM | *P* values |
| --- | --- | --- | --- | --- | --- | --- |
|  |  | 2% | 4% | 6% |  |  |
| Days 1−14 of the trial | 14.95 | 11.83 | 15.18 | 20.76 | 1.63 | 0.28 |
| Days 15−28 of the trial | 2.53 | 1.34 | 1.86 | 3.57 | 0.64 | 0.65 |
| Days 29−42 of the trial | 1.56 | 1.79 | 1.19 | 2.01 | 0.38 | 0.90 |
| Days 1−42 of the trial | 6.06 | 4.99 | 6.26 | 8.78 | 0.71 | 0.31 |

Data are presented as means with pooled SEM (*n* = 8).
